# Comment on "Increased risk of second cancers at sites associated with HPV after a prior HPV-associated malignancy, a systematic review and meta-analysis"

**DOI:** 10.1038/s41416-019-0439-0

**Published:** 2019-03-26

**Authors:** Hélène Péré, Juliette Pavie, Simon Pernot, David Veyer, Dominique Bertaud, Sophie Hurel, Laurent Bélec, Stéphane Hans, Madeleine Ménard, Béatrix Cochand-Priollet, Laurence Weiss, Anne-Sophie Bats, Cécile Badoual, AP-HP CoMPap (Consultation Multidisciplinaire Papillomavirus) group

**Affiliations:** 1 Laboratoire de virologie, Hôpital Européen Georges Pompidou, Assistance Publique-Hôpitaux de Paris, Paris, France; 2 Faculté de Médecine Paris Descartes, Université Paris Descartes, Sorbonne Paris Cité, Paris, France; 3 Service d’immunologie Clinique, Hôpital Européen Georges Pompidou, Assistance Publique-Hôpitaux de Paris, Paris, France; 4 Service d’oncologie digestive, Hôpital Européen Georges Pompidou, Assistance Publique-Hôpitaux de Paris, Paris, France; 5 Laboratoire d’anatomo-cytopathologie, Hôpital Européen Georges Pompidou, Assistance Publique-Hôpitaux de Paris, Paris, France; 6 Service d’urologie, Hôpital Européen Georges Pompidou, Assistance Publique-Hôpitaux de Paris, Paris, France; 7 Service d’ORL et de chirurgie de la tête et du cou, Hôpital Foch, Suresnes, France; 8 Service d’ORL et chirurgie cervico-faciale, Hôpital Européen Georges Pompidou, Assistance Publique-Hôpitaux de Paris, Paris, France; 9 Laboratoire d’anatomo-cytopathologie, Hôpital Cochin, Assistance Publique-Hôpitaux de Paris, Paris, France; 10 Service de chirurgie cancérologique et gynécologique Hôpital Européen Georges Pompidou, Assistance Publique-Hôpitaux de Paris, Paris, France

**Keywords:** Cancer prevention, Outcomes research

We read with a great interest the meta-analysis of Gilbert D.C. et al. recently published in *British Journal of Cancer*^1^ and contributing to the evaluation of the potential risk of HPV-related second cancer in patients with a history of a primary HPV-related cancer in the same or an independent mucosal site.

Oncogenic high-risk HPV (HR-HPV) are involved in about 5% of overall cancers^2^ and it is now well established that HPV infection is involved in the oncogenesis of genital (cervix, vulva, vagina, penis and anus) or oropharyngeal carcinomas. Patients with primary HPV squamous cell carcinoma may have increased risk to develop HPV-related second malignancies, in the same or in another mucosal site.^3–8^ However, the vast majority of available data regarding the risk of developing secondary cancers or high-grade intraepithelial lesions for a patient who has presented an index HPV-induced cancer originate from retrospective studies essentially focused on two mucosal sites^1^ and as it was discussed by Gilbert et al., all of these retrospective studies are very heterogenous.

Even if the tendency towards an increased risk was observed in all studies and across each of the mucosal sites, it is still difficult to clearly recommend a systematic follow-up for these patients and to define how this monitoring should be carried out. Indeed, the studies are very heterogenous and present clear limitation due to their retrospective design, especially for the ones which reported rates of a second independent cancer after an initial diagnosis of primary HPV-associated cancer.

The European Georges Pompidou Hospital (AP-HP Paris, France) takes advantage of a large recruitment of patients suffering from HPV-related cancer (ano-genital and head and neck cancers), as well as HPV biological and clinical experts. Diagnosing and treating HPV-induced cancers represents a unique opportunity to prevent subsequent HPV-associated cancers with a multidisciplinary follow-up. We have created in 2014, for the first time to our knowledge, the so-called CoMPap (Consultation Multidisciplinaire Papillomavirus) HPV multidisciplinary consultation based on an annual prospective monitoring of patients with a history of HPV-related primary tumour. To be enroled, the patients have to sign a dedicated informed consent approved by the ethical committee.

Included patients are annually examined by clinicians (gynaecologist/proctologist/urologist/ear, nose and throat specialists) to detect macroscopic mucosal lesion in all HPV-concerned mucosal site. In parallel, pap-smears are collected for cytological and virological evaluation. Different infection sexually transmitted serological status are checked and a psychologist can also be requested for patient care if indicated. Finally, the CoMPap consultation allows an innovative medical care of the HPV cancers allying supervision, prevention, psychological support and biobanking.

Until now, 59 patients (median age 55 years) have been enroled in the CoMPap. The cohort includes a majority of women (*n* = 41; 69.5%) essentially enroled after in situ or infiltrative genital carcinoma diagnosis. Eighteen men were also included and monitored.

Remarkably, at baseline, 19% of enroled patients had already presented a history of an index HPV-associated squamous cell carcinoma between 6 and 24 years before the mucosal HPV cancer leading them to be enroled in the CoMPap. As a first result, this percentage seems to be in agreement with the observation of Gilbert D.C. et al. in their meta-analysis, the risk of second HPV-associated cancer for patients diagnosed with HPV-associated tumoural or pre-tumoural lesions was estimated to be around a five-fold increase as compared with unaffected patients.

Our preliminary observations, confirming and extending the retrospective results reported by Gilbert D.C. et al. about the increased risk of developing secondary HPV-associated tumours in patients with a history of HPV-related cancer, seem to support the informative value of the CoMPap implemented at the European Georges Pompidou Hospital (AP-HP Paris, France) to closely monitor HPV-related cancer patients in prospective cohorts to prevent or diagnose at an earlier curable stage of these potential secondary malignancies.
